# Hypothalamic Signaling Mechanisms in Hypertension

**DOI:** 10.1007/s11906-015-0550-4

**Published:** 2015-04-10

**Authors:** Casey Y. Carmichael, Richard D. Wainford

**Affiliations:** Department of Pharmacology & Experimental Therapeutics and The Whitaker Cardiovascular Institute, Boston University School of Medicine, 72 East Concord St, Boston, MA 02118 USA

**Keywords:** Hypothalamus, Hypertension, Signal transduction, Paraventricular nucleus, Arcuate nucleus, Hypertension, Autonomic control

## Abstract

The etiology of hypertension, a critical public health issue affecting one in three US adults, involves the integration of the actions of multiple organ systems, including the central nervous system. Increased activation of the central nervous system, driving enhanced sympathetic outflow and increased blood pressure, has emerged as a major contributor to the pathogenesis of hypertension. The hypothalamus is a key brain site acting to integrate central and peripheral inputs to ultimately impact blood pressure in multiple disease states that evoke hypertension. This review highlights recent advances that have identified novel signal transduction mechanisms within multiple hypothalamic nuclei (e.g., paraventricular nucleus, arcuate nucleus) acting to drive the pathophysiology of hypertension in neurogenic hypertension, angiotensin II hypertension, salt-sensitive hypertension, chronic intermittent hypoxia, and obesity-induced hypertension. Increased understanding of hypothalamic activity in hypertension has the potential to identify novel targets for future therapeutic interventions designed to treat hypertension.

## Introduction

Hypertension, a significant public health issue, affects one third of the US adult population and is a critical risk factor for future adverse cardiovascular events—significantly contributing to the burden of cardiovascular and kidney disease, stroke, and premature death and disability [1]. As a heterogenous disease, the pathogenesis of hypertension has remained elusive but likely involves the complex integration of multiple regulatory systems. Recent work has explored the role of brain mechanisms in both the short- (i.e., initiation) and long-term (i.e., maintenance) control of blood pressure as increasing evidence implicates activation of the central nervous system as a key contributor to the development of hypertension [2]. The hypothalamus is an essential component in the regulatory neural network of central blood pressure control serving to coordinate and integrate signal transduction in response to central and peripheral stimuli [3]. The hypothalamus acts as the interface between the endocrine and nervous systems, and a wide variety of functional changes in the hypothalamus characterize multiple forms of hypertension. Highlighting the importance of central pathways regulating blood pressure, it has been suggested that more than 50 % of clinical hypertension cases can be categorized as neurogenic essential hypertension [4]. Therefore, a more detailed understanding of dysfunctional reflexes and/or altered neural circuitry within the hypothalamus will potentially reveal novel targets for future therapeutic intervention to treat hypertension. The purpose of this review is to highlight recent advancements in our understanding of signal transduction pathways within the hypothalamus that impact the etiology and pathogenesis of hypertension.

## Advances in Hypothalamic Neuronal Signaling in Hypertension

Increased neuronal activity and excitability (i.e., synaptic plasticity) in the hypothalamic paraventricular nucleus (PVN) mediates elevated sympathetic drive in multiple hypertensive animal models. Recent work has focused on identifying the synaptic mechanisms driving increased PVN neuronal activity. In the Spontaneously Hypertensive Rat (SHR) model of neurogenic hypertension, patch-clamp electrophysiological studies conducted in brain slices have revealed that there is increased activity of spinally projecting PVN presympathetic neurons [5–7] and preautonomic PVN neurons that innervate the nucleus tractus solitarius [8]. Increased excitability of preautonomic PVN neurons is primarily mediated by an input-output function in response to depolarizing stimuli [8]. PVN presympathetic neuron tone is regulated by excitatory and inhibitory GABAergic inputs. The polarities of the actions of γ-aminobutyric acid (GABA) on the GABA_A_ receptor, a ligand-gated ion channel, are strongly influenced by intracellular chloride concentrations. In the SHR, dysregulation of the intracellular chloride homeostasis of PVN presympathetic neurons is evoked by upregulation and glycosylation of the Na^+^-K^+^-2Cl^−^ cotransporter 1 (NKCC1) [5], and this contributes to the impaired PVN GABAergic synaptic inhibition and increased sympathetic tone observed in the SHR. Additionally, impairment of PVN GABAergic inhibitory signaling has been observed in vivo in a mouse model of neurogenic hypertension (the Schlager mouse). This alteration of GABAergic signaling is postulated to contribute to Schlager mouse hypertension through reduced PVN inhibitory drive to presympathetic neurons [9].

Highlighting the complexity of PVN glutamatergic pathways on the activity of spinally projecting PVN neurons are the opposing actions of group I and group II metabotropic glutamate receptors (mGluRs). The activity of group II mGluRs at PVN glutamatergic terminals, determined via patch-clamp studies in isolated brain slices, is enhanced in the SHR [6]. This elevated activation of mGluRs attenuates increased glutamatergic input via a negative feedback pathway to reduce presympathetic PVN neuronal hyperactivity. In companion in vivo PVN microinjection studies, mGluR activation evoked reductions in sympathetic vasomotor tone and blood pressure in the SHR [6], suggesting that activation of PVN group II mGluRs attenuates elevated glutamateric inputs and neuronal hyperactivity in the SHR. In contrast, determined in hypothalamic slices, increased endogenous expression of the group I mGluR5 in the PVN of the SHR drives increased basal firing of spinally projecting PVN neurons [7]. In vivo studies provide evidence that the mechanism underlying mGluR5-driven hyperactivity of PVN presympathetic neurons in the SHR involves recruitment of *N*-methyl-d-aspartate (NMDA) receptors to the cell surface. This occurs via a protein kinase C (PKC) and synaptosomal-associated protein-mediated signal transduction pathway to increase NMDA receptor-mediated activity and subsequently sympathetic tone and blood pressure [7].

Increased presympathetic PVN neuron firing in the SHR, assessed in hypothalamic slices, is driven in part by an NMDA receptor-calpain-calcineurin signaling pathway that evokes glutamate receptor 2 (GluR2) internalization, determined in vivo, increasing the activity of GluR2-lacking α-Amino-3-hydroxy-5-methyl-4-isoxazolepropionic acid (AMPA) receptors [10]. In addition to altering AMPA receptor activity, the NMDA receptor directly increases glutamatergic activity in the SHR. Potentiated pre- and postsynaptic PVN NDMA receptor activity, determined via brain slice electrophysiological studies, is mediated by upregulation of synaptic GluN2A, an NMDA receptor subunit, that is dependent on PKCK2 activity [11]. PKCK2 also influences the activity of the small conductance calcium-activated K^+^ (SK) channel in the PVN. The SK channel impacts the intrinsic activity of presympathetic PVN neurons, and SK activity is reduced in the SHR via a CK2-mediated calmodulin phosphorylation mechanism [12]. Clinical trials are currently investigating the safety of CK2 inhibitors, which may have a future therapeutic application to reduce sympathetic outflow via the modulation of the activity of SK channels and NMDA receptors. A critical issue facing the field, which remains to be fully addressed, is the potential difference in synaptic activity in vivo versus the measurements made in brain slices in which synaptic connections and synaptic drive are lost. Owing to considerable technical impediments, despite the significant insight provided via electrophysiological patch-clamp studies conducted in PVN neuron containing brain slices [5–8, 10–12], our understanding of PVN neuronal synaptic activity in vivo in hypertensive animals models remains limited.

The hypothalamic arcuate nucleus (ARCN) dramatically impacts cardiovascular function and sympathetic outflow in vivo [13, 14, 15•]. At normal blood pressures, NMDA activation of the ARCN in Wistar rats evokes decreases in blood pressure and splanchnic nerve activity mediated by PVN GABA_A_, neuropeptide Y1, and opioid receptors [13]. Significantly, if blood pressure is lowered, NMDA stimulation of the ARCN evokes increases in blood pressure mediated by PVN melanocortin receptors [13]. In the Wistar rat, barodenervation studies reveal that interruption of the baroreceptor afferents is responsible for converting NMDA-evoked melanocortin and ionotropic glutamate receptor mediated ARCN cardiovascular and sympathetic depressor actions into pressor responses [14]. In the baro-intact hypertensive SHR, which exhibits attenuated ARCN baroreceptor and GABAergic function [15•], NMDA activation of the ARCN evokes elevations in blood pressure and splanchnic nerve activity, as observed in the barodenervated Wistar rat [15•]. Collectively, these data highlight the previously unknown role of the ARCN in the regulation of blood pressure and sympathetic activity in normotensive and hypertensive phenotypes in vivo. The potential impact of ARCN signal transduction in other cardiovascular disease states featuring increased blood pressure (e.g., obesity, salt-sensitive hypertension) remains to be established.

## Advances in Hypothalamic Inflammatory Pathways in Hypertension

Increased hypothalamic expression of proinflammatory cytokines (PIC) is closely associated with the pathophysiology of hypertension. In particular, multiple studies have demonstrated an interaction between the renin-angiotensin system (RAS)-mediated hypertension and inflammation [16]. Nuclear factor-κB (NFκB) is a key transcription factor governing inflammatory processes and plays a critical role in mediating inflammation-induced hypertension. Blockade of NFκB within the PVN significantly attenuates angiotensin II (Ang II)-induced hypertension—a response accompanied by a reduction in PIC and upregulation of the protective anti-hypertensive arms of the RAS system [17]. A subsequent study revealed PVN specific blockade of tumor necrosis factors (TNF) significantly attenuates Ang II-evoked hypertension and increases in PIC and prevented Ang II-evoked decreases in pro-hypertensive RAS components. This highlights a key role of TNF in Ang II-stimulated inflammation [18]. Illustrating the cross talk between reactive oxygen species (ROS) and PIC signal transduction pathways, central administration of the ROS scavenger tempol attenuates Ang II increases in PVN interleukin (IL) 1β, sympathetic activity, and blood pressure [19]. Despite the clear involvement of Ang II in stimulating PIC, the pathways that initiate this response remain to be fully defined. Disruption of PVN p44/42 mitogen-activated protein kinase (MAPK) signal transduction during the early phase of slow pressor Ang II infusion, prior to the development of hypertension, attenuates Ang II hypertension and sympathoexcitation, but does not reduce Ang II-evoked increases in PIC [20]. In the deoxycorticosterone acetate-salt (DOCA-salt) model of neurogenic hypertension, overexpression of angiotensin-converting enzyme 2 (ACE2) attenuates hypertension and reverses DOCA-salt-induced increases in PVN extracellular signal regulated kinase (ERK) and cyclooxygenase 1-mediated inflammation [18]. This suggests a role for ERK pathways in the Ang II-evoked inflammatory pathway that contributes to hypertension.

Recent evidence supports a novel direct action of prorenin, another key component of the RAS system, to activate PVN microglia that drives proinflammatory signaling and hypertension [21]. The actions of Ang II to drive inflammation also occur in obesity-induced hypertension, a complex model of hypertension, in which PVN-specific angiotensin type 1 receptor (AT1_A_R) deletion attenuates hypothalamic inflammation and high-fat diet induced increases in blood pressure [22]. In contrast to the body of evidence presented above, it was recently reported that model that blockade of PVN TNFα does not reduce blood pressure or sympathetic nerve activity in the Ang II-salt hypertension [23]. These conflicting data highlight that our understanding of the role of inflammation in the regulation of blood pressure is far from complete.

## Advances in Hypothalamic Oxidative Stress Pathways in Hypertension

Within the PVN, ROS modulates sympathetic nerve activity and the development of hypertension. Virally mediated overexpression of PVN superoxide dismutase (SOD1) significantly attenuates hypertension, sympathetic activity, and the cardiac sympathetic afferent reflex (CSAR) in the SHR rat [24]. PVN-specific inhibition of ROS, achieved by bilateral PVN infusion of the free radical scavenger tempol, attenuates intravenous Ang II-induced hypertension and renal sympathoexcitation [19]. Further, abolishment of Ang II mediated increases in PVN ROS, and blood pressure are observed following PVN-specific NFκB inhibition [17]. Both of these studies [17, 19] support the hypothesis that Ang II-generated intra-PVN ROS contributes to the pathophysiology of Ang II hypertension. During slow pressor Ang II hypertension, increased membrane mobilization of the nicotinamide adenine dinucleotide phosphate oxidase subunit (NOX) p47^phox^ correlates with increased ROS production in PVN neurons. This suggests an influence of Ang II to drive mobilization of cytoplasmic nicotinamide adenine dinucleotide phosphate (NADPH) p47^phox^ stores to increase oxidative stress [25]. Additionally, slow pressor Ang II-mediated production of ROS reduces PVN nitric oxide (NO) signal transduction and increases glutamatergic signaling—a change postulated to contribute to neural dysfunction in this model of hypertension [25]. Interestingly, the temporal impact of changes in NO signaling in the pathophysiology of hypertension remains relatively unexplored. Partially addressing this knowledge gap, it has been reported that reductions in PVN NO activity that occur during the onset of hypertension in the renal wrap hypertensive rat model may act as a trigger event for the development of hypertension [26].

Improved NO signaling attenuates hypertension in animal models—experimental data that demonstrate that exploiting the anti-hypertensive actions of NO represents a potential treatment modality for hypertension. In the 2-kidney 1-clip (2K1C) rat, activation of PVN adrenomedullin receptors by exogenous intermedin evokes decreased renal sympathetic nerve activity and long-term reductions in the magnitude of hypertension by increasing the availability of NO metabolites [27]. In DOCA-salt hypertension, neuron-targeted overexpression of ACE2 increases PVN levels of SOD and manganese SOD and attenuates DOCA-salt-evoked oxidative stress leading to attenuation of neurogenically mediated hypertension [18]. The recent advances detailed above highlight the involvement of oxidative stress pathways in multiple forms of hypertension, and suggest that further investigation of these mechanisms may result in the discovery of new therapeutic avenues for hypertension treatment.

## Advances in Hypothalamic Regulation of Vasopressin Secretion in Hypertension

Although the role of arginine vasopressin (AVP) in hypertension remains under debate, increasing evidence implicates elevated AVP in the pathogenesis of several forms of human hypertension. Recent studies in the stroke-prone SHR correlated increased PVN and supraoptic nucleus (SON) magnocellular AVP levels with both age and magnitude of hypertension [28]. Nonetheless, the mechanisms by which AVP levels are increased to drive or maintain hypertension remain poorly defined. It has been hypothesized that in the SHR, elevated hypothalamic magnocellular expression of the mineralocorticoid receptor (MR) drives AVP release to contribute to the maintenance of hypertension—a theory supported by MR antagonism normalizing blood pressure in the SHR phenotype [29]. Further, in the 2K1C model of renovascular hypertension, AVP contributes to hypertension via a PVN-specific salusin-β AVP-V1 receptor-dependent (Ang II-independent) pathway [30]. This evokes increased sympathetic activation, and subsequently hypertension, via downstream signal transduction and V1 receptor activation in the rostral ventrolateral medulla (RVLM) [30]. Extending the involvement of AVP in hypertension are recent investigations reporting that impaired GABAergic inputs to PVN and SON vasopressin neurons may contribute to the development of salt-sensitive hypertension. In the DOCA-salt rat model, a gradual and progressive “inhibitory-to-excitatory” switch in the GABA_A_ receptor state occurs, mediated by depolarization of the GABA equilibrium potential. This switch is dependent on altered intracellular chloride homeostasis in AVP neurons that is driven by upregulation of NKCC1 and downregulation of the K^+^-Cl^−^ cotransporter isotype 2 (KCC2) [31]. This transforms the canonical inhibitory GABA_A_ receptor response into a stimulatory drive in hypothalamic magnocellular AVP neurons to increase AVP release—a switch (i.e., excitatory GABAergic input) that may drive the development of salt-sensitive hypertension [31]. Downregulation of PVN KCC2 is also evoked during high salt intake by brain-derived neurotrophic factor (BDNF) activation of a tyrosine kinase receptor B (TrkB) pathway [32•]. KCC2 downregulation alters the chloride gradient across SON neurons, abolishing aortic baroreceptor-evoked GABAergic inhibition of AVP neurons. This alteration in inputs to AVP neurons drives excessive AVP release to trigger hypertension via peripheral V1 receptor-mediated vasoconstriction [32•]. Collectively, these studies provide insight into AVP signal transduction within the PVN and SON and suggest multiple therapeutic targets to reduce AVP release in hypertension.

## Advances in Hypothalamic Signaling in Salt-Sensitive Hypertension

Excess dietary salt intake is an established cause of hypertension resulting in an increased risk of adverse cardiovascular events [33, 34]. At present, the mechanism(s) by which dietary salt evokes hypertension remain to be fully elucidated. In the Dahl salt-sensitive (DSS) rat phenotype, sympathoexcitatory and pressor responses to increased cerebrospinal fluid (CSF) sodium are enhanced [35]. In salt-resistant rat phenotypes, a hypothalamic intraneuronal salt-inducible kinase 1 (SIK1)-N^+^/K^+^ATPase activity pathway reduces CSF sodium-induced sympathoexcitation and increases in blood pressure [35]. In contrast, in the DSS rat, reduced hypothalamic SIK1 expression and activity suggests this anti-hypertensive mechanism is downregulated and may contribute to salt-sensitivity [35]. An additional mechanism contributing to the maintenance of elevated blood pressure in hypertensive DSS rats involves activation of both PVN glutamate and angiotensin type 1 receptors (AT_1_R)—a response that involves enhanced AT_1_R-mediated local glutamate receptor activation [36] and is absent in normotensive DSS and Dahl salt-resistant (DSR) rats. Further, a role of increased brain MR receptor activation has been suggested to drive the activation of PVN glutamate and AT_1_R in Ang II hypertension in the Wistar rat [36].

It is well documented that chronic sodium intake alters neuronal activity of the PVN; however, the temporal dependency of these changes remains unclear. Acute activation of forebrain sympathoexcitatory osmosensory inputs to the PVN, via acute internal carotid artery infusion of hypertonic saline and activation of glutamatergic pathways, rapidly increases splanchnic and phrenic nerve activity and blood pressure [37]. These studies demonstrate that acute sodium-evoked activation of the PVN is able to drive increased vasomotor sympathetic nerve activity and blood pressure, suggesting that PVN neural activation may be an early event in the pathophysiology of salt-sensitivity. During chronic high salt intake, the forebrain hypothalamus also plays a critical role in modulating central sympathetic outflow [38]. Highlighting the adverse impact of excess salt intake, forebrain hypothalamic circuitry mediates sodium-evoked sensitization of sympathetic responses and increased blood pressure variability in salt-resistant Sprague–Dawley (SD) rats [38].

A recent focus has been on the identification of potential mechanisms maintaining salt-resistance [39–41]. In the salt-resistant SD rat, in response to changes in dietary sodium intake, there is an endogenous PVN-specific increase (sodium excess) or decrease (sodium restriction) in guanine nucleotide binding protein alpha inhibiting activity binding polypeptide 2 (Gαi_2_) protein expression [39]. Acute and chronic downregulation of brain Gαi_2_ proteins in high salt-challenged SD rats, which prevents endogenous PVN Gαi_2_ protein upregulation, evokes sodium retention, global sympathoexcitation, and elevated blood pressure [39, 40] suggesting a critical role of these proteins in maintaining salt-resistance. An endogenous sodium-evoked increase in PVN Gαi_2_ protein expression has been documented in multiple salt-resistant phenotypes, as has a failure of this response in the DSS rat [41]. Further, brain Gαi_2_ proteins are required to maintain salt-resistance, via a renal nerve-dependent pathway, in the salt-resistant SD and DSR rat [40, 41]. Collectively, PVN-specific Gαi_2_-subunit protein–gated signal transduction pathways represent a novel central molecular signal transduction pathway that acts to regulate central sympathetic outflow to the kidneys in response to alterations in salt intake to maintain a salt-resistant phenotype [39–41].

## Advances in Hypothalamic Pathways Involved in Chronic Intermittent Hypoxia

Chronic intermittent hypoxia (CIH), an animal model of human sleep apnea, exhibits increased blood pressure and elevated sympathetic tone as is observed in the clinical phenotype. Central administration of losartan attenuates the sustained component of CIH-induced hypertension [42]. Blockade of the actions of Ang II decreases FosB/ΔFosB staining in the parvocellular regions of the PVN (and other autonomic control sites), indicating that brain RAS contributes to increased transcriptional activation in autonomic nuclei to drive CIH-mediated hypertension [42]. During CIH, the contribution of PVN neuronal activity to support lumbar sympathetic nerve activity (LSNA) and blood pressure is increased, as is the responsiveness of PVN-mediated increases in LSNA to hypertonic saline [43]. Further supporting a central role of the PVN in CIH-evoked sympathoexcitation is evidence that acute and chronic CIH modulates the excitatory output from the PVN to brain stem parasympathetic vagal neurons, which leads to a reduction in PVN-evoked excitatory glutamatergic postsynaptic currents. The resulting diminished activation of cardiac vagal neurons potentially increases the risk of adverse cardiovascular events during episodes of sleep apnea [44]. These data suggest that CIH-mediated hypertension features impairments in both sympathetic and parasympathetic tone evoked by altered PVN neuronal activity [43, 44]. A recent clinical study provided evidence that sleep apnea patients’ elevated spontaneous muscle sympathetic nerve activity is related to functional changes in activity of several cortical and subcortical regions, but did not report changes in activity of the hypothalamus [45]. This discrepancy may reflect differences between animal models of CIH and the clinical phenotype.

## Advances in Hypothalamic Pathways Activated in Obesity-Induced Hypertension

The hormone leptin, released from white adipose tissue, acts within the hypothalamus via intracellular AMP-activated protein kinase (AMPK) signaling pathways to evoke sympathoexcitation (particularly to the kidney) and hypertension [46]. Recent discoveries have determined that hypothalamic AMPKα2 mediates the hypertensive and regional (renal and adipose) sympathoexcitation evoked by central leptin [46]. AMPKα2 therefore plays a previously unknown role in mediating leptin-evoked increased sympathetic stimulation of both the kidneys and adipose tissue. Extending this novel role of leptin, it has been demonstrated that leptin also activates the mammalian target of rapamycin (mTORC1) via a phosphoinositide 3-kinase (PI3K) pathway [47•]. Further, mTORC1 activity is required to mediate leptin-induced increases in renal sympathetic nerve activity (RSNA) and blood pressure via a mechanism lying upstream of the melanocortin receptor system [47•]. Within the hypothalamus, ARCN-specific blockade of mTORC1 signaling decreases baseline RSNA and blood pressure and essentially eliminates leptin-evoked hypertension and renal sympathoexcitation [47•].

Recent evidence has revealed the existence of striking sex differences in leptin’s ability to increase sympathetic nerve activity and blood pressure. The stimulatory effects of leptin on blood pressure are absent in female rats [48]. Additionally, leptin-evoked increases in LSNA and RSNA are only evident during pro-oestrus and estrogen-treated ovariectomized female rats, and are mediated via a mechanism involving increased alpha-melanocyte-stimulating hormone activation of PVN presympathetic neurons [48]. Beyond direct sex differences, it has been documented that maternal obesity (triggered by a high-fat diet) results in the offspring of rabbits exhibiting increased blood pressure and RSNA [49] via a mechanism involving increases in hypothalamic sensitivity to leptin [49]. Collectively, these findings suggest that it may be beneficial to consider both gender and maternal environment in future studies into the mechanisms of obesity-induced hypertension.

A direct role of insulin signaling in cardiovascular dysregulation resulting in hypertension in obesity and/or diabetes remains controversial. However, the ARCN has been identified as a key site at which insulin evokes increases in LSNA [50]. Insulin-mediated stimulation of the ARCN increases blood pressure and LSNA via a downstream pathway requiring glutamate receptor activation in the PVN. Recently, it has been documented via direct recordings of LSNA that activation of PVN glutamate receptors contributes to insulin-evoked sympathoexcitation through an NMDA-dependent signal transduction pathway [51]. Further, highlighting the importance of mTORC1 and PI3K signaling pathways in the regulation of sympathetic nerve activity, insulin evokes activation of LSNA via a hypothalamic ARCN PI3K-mTORC1 pathway [52, 53]. The sympathoexcitatory actions of insulin, centrally or locally within the ARCN, are attenuated by glucocorticoid administration [52] via pathway(s) located downstream of the above-described PI3K-mTORC pathway. These molecular studies provide new targets for therapeutic compounds to treat the sympathetic overactivity and hypertension associated with obesity.

## Novel Paradigms in Hypothalamic Signaling in Hypertension

### Blood Brain Barrier Disruption

Ang II activates a multitude of hypothalamic signal transduction pathways to drive the pathophysiology of hypertension. However, how circulating Ang II exerts central actions has long been under debate, owing to the fact that under normal physiological conditions, Ang II is unable to penetrate the blood brain barrier (BBB). Recent data demonstrate that Ang II impairs the integrity of the BBB in the hypothalamic region via an AT_1_R mechanism in the SHR. This facilitates elevated Ang II levels and Ang II co-localization with neurons and glial in the PVN—sites normally protected from the actions of circulating Ang II [54••]. These data provide the first evidence that circulating Ang II, in a hypertensive condition, exerts direct actions in the brain in a hypertensive setting, beyond the established actions on projections emanating from the subfornical organ that drive neurohumoral activation in hypertension. It remains to be established if Ang II disrupts BBB integrity to drive central Ang II signal transduction and increases in blood pressure in additional hypertensive models.

### Central Nervous System Sensitization

Recent evidence highlights a direct role of central nervous system sensitization in the pathogenesis of Ang II slow pressor hypertension via an AT_1_R mechanism [55••]. Seven days of sensitization with a non-pressor dose of Ang II, administered centrally or peripherally, dramatically exacerbates the subsequent hypertensive response to slow pressor Ang II infusion [55••]. Sensitization evokes increased PVN MR receptor expression and PVN neuronal activation during slow pressor Ang II infusion. Highlighting the interaction between aldosterone and the RAS, subpressor aldosterone (centrally or peripherally) cross-sensitizes and exacerbates slow pressor Ang II hypertension via a PVN MR receptor pathway [55••]. These data highlight the impact of hypothalamic neuroplasticity on the pathophysiology of hypertension—a comparatively unexplored field that will likely yield valuable insights into the central regulation of blood pressure.

### Anterior Hypothalamic Parvalbuminergic Neurons

A previously unknown population of parvalbuminergic neurons, which require thyroid hormone signaling for proper development, has been identified in the anterior hypothalamus (AH) [56••]. Significantly, this neuronal population has a profound impact on blood pressure regulation as ablation of these cells evokes hypertension. The mechanism involved in increasing blood pressure following ablation of AH parvalbuminergic neurons occurs independently of alterations in the RAS and appears to be mediated by alterations in central autonomic function [56••]. Due to the recent discovery of this neuronal population, the potential impact of this AH parvalbuminergic cell population in hypertension remains to be fully defined, as does the impact of thyroid disease on blood pressure regulation.

## Conclusion

Over the last several years, our understanding of the signaling pathways acting within the hypothalamus to regulate blood pressure has dramatically increased. The PVN remains a predominant site within the hypothalamus at which signal transduction pathways in multiple hypertensive disease states evoke autonomic and endocrine imbalance to drive elevations in both sympathetic outflow and blood pressure. An increasingly important role of the ARCN in blood pressure regulation and renal nerve activity has been revealed, suggesting this hypothalamic nucleus may play a larger than previously thought role in the long-term regulation of blood pressure. The next few years will provide further delineation of hypothalamic disease-specific signaling pathways and may identify potential cross talk between these mechanisms across hypertension disease states. We speculate that current and future identification of hypothalamic signaling mechanisms will generate therapeutic targets with broad applications to treat human hypertension.
